# Recombinant human thrombopoietin therapy for primary immune thrombocytopenia in pregnancy: a retrospective comparative cohort study

**DOI:** 10.1186/s12884-023-06134-y

**Published:** 2023-11-27

**Authors:** Jing Lin, Tong-Fei Wang, Mei-Juan Huang, Hao-Bo Huang, Pei-Fang Chen, Yu Zhou, Wei-Chao Dai, Ling Zhou, Xiu-Shan Feng, Hui-Lan Wang

**Affiliations:** 1 Department of Ob and Gyn, Fujian Medical University Union Hospital, Fuzhou, China; 2 Fujian Institute of Haematology, Fujian Provincial Key Laboratory of Haematology, Fujian Medical University Union Hospital, Fuzhou, China; 3 Department of Blood Transfusion, Fujian Medical University Union Hospital, Fuzhou, China

**Keywords:** Recombinant human thrombopoietin, Primary immune thrombocytopenia, Pregnancy, Generalised estimating equation

## Abstract

**Background:**

Treatment options for pregnant women with immune thrombocytopenia (ITP) who do not respond to first-line treatment are limited. Few studies have reported the use of recombinant human thrombopoietin (rhTPO) for this subset of patients.

**Aims:**

To investigate the efficacy and safety of rhTPO in ITP during pregnancy and determine obstetric outcomes and predictors of treatment response.

**Methods:**

From July 2013 to October 2022, the data of 81 pregnant women with ITP and a platelet count < 30 × 10^9^/L who did not respond to steroids and/or intravenous immunoglobulin were retrospectively analysed. Of these patients, 33 received rhTPO treatment (rhTPO group) while 48 did not (control group). Baseline characteristics, haematological disease outcomes before delivery, obstetric outcomes, and adverse events were compared between groups. In the rhTPO group, a generalised estimating equation (GEE) was used to investigate the factors influencing the response to rhTPO treatment.

**Results:**

The baseline characteristics were comparable between both groups (*P* > 0.05, both). Compared with controls, rhTPO patients had higher platelet counts (median [interquartile range]: 42 [21.5–67.5] vs. 25 [19–29] × 10^9^/L, *P* = 0.002), lower bleeding rate (6.1% vs. 25%, *P* = 0.027), and lower platelet transfusion rate before delivery (57.6% vs. 97.9%, *P* < 0.001). Gestational weeks of delivery (37.6 [37–38.4] vs 37.1 [37–37.2] weeks, *P* = 0.001) were longer in the rhTPO group than in the control group. The rates of caesarean section, postpartum haemorrhage, foetal or neonatal complications, and complication types in both groups were similar (all *P* > 0.05). No liver or renal function impairment or thrombosis cases were observed in the rhTPO group. GEE analysis revealed that the baseline mean platelet volume (MPV) (odds ratio [OR]: 0.522, *P* = 0.002) and platelet-to-lymphocyte ratio (PLR) (OR: 1.214, *P* = 0.025) were predictors of response to rhTPO treatment.

**Conclusion:**

rhTPO may be an effective and safe treatment option for pregnancies with ITP that do not respond to first-line treatment; it may have slightly prolonged the gestational age of delivery. Patients with a low baseline MPV and high baseline PLR may be more responsive to rhTPO treatment. The present study serves as a foundation for future research.

## Background

Primary immune thrombocytopenia (ITP) is an acquired autoimmune haemorrhagic disease, with an incidence during pregnancy ranging from 1/1000 to 1/10000 [1–3]. Among patients with pre-existing ITP, platelet counts may be further reduced during pregnancy [4], increasing bleeding risk [2, 5]. Treatment aims to raise the platelet count above 20 × 10^9^/L during pregnancy and 50 × 10^9^/L at delivery to minimise bleeding risk [6]. Steroids and/or intravenous immunoglobulins (IVIG) are considered first-line treatments [1, 7]. However, the response rate of pregnant patients with ITP to either steroids or IVIG is reportedly less than 40% [8, 9]. Treatment options for ITP during pregnancy are limited if the patient does not respond to steroids and/or IVIG. Splenectomy during pregnancy is associated with increased foetal risk and technical difficulties. The effects of drug administration on the foetus during gestation should be considered. Azathioprine and rituximab should be avoided whenever possible [1, 6]. TPO-RAs may be considered in exceptional circumstances, ideally only in the third trimester near delivery [6]. Platelet transfusions are usually associated with a poor increment and rapid clearance, with alloimmunistation following repeated transfusion further reducing responses. Response to any platelet transfusion in ITP must be closely monitored and reserved for instances where urgent elevation of the platelet count is required in the face of treatment failures. Thus, treatment options represent a difficult problem for women who do not respond to first-line treatment.

In China, recombinant human thrombopoietin (rhTPO), a glycosylated full-length thrombopoietin (TPO) expressed by Chinese hamster ovary cells using gene recombination followed by purification, exhibits biological functions similar to those of endogenous TPO [10]. TPO receptor (TpoR) is expressed in bone marrow haematopoietic stem cells, megakaryocytes, and platelet membranes. rhTPO promotes megakaryocyte proliferation, differentiation, and maturation, and platelet generation by binding to TpoR [10, 11]. This drug is approved by the China Food and Drug Administration for treating patients with chronic immune-, chemotherapy-related, or septic thrombocytopenia who do not respond to first-line therapy [10].

Animal experiments [11] have shown that rhTPO has no teratogenic effects. A small-sample single-arm study reported that 31 pregnant women with ITP whose platelet count was lower than 30 × 10^9^/L and who did not respond to steroids or IVIG had an overall response rate of 74% to rhTPO with no adverse effects. Simultaneously, the study has shown that rhTPO cannot cross the placenta [4]. However, the study lacked a control group; thus, the evidence provided is limited. No study has compared rhTPO treatment with no treatment (active surveillance) in pregnant patients with ITP who do not respond to first-line treatment. Moreover, the clinical predictors associated with the therapeutic effects of rhTPO remain unclear. Studies have demonstrated a correlation of mean platelet volume (MPV) [12, 13] and platelet-to-lymphocyte ratio (PLR) [14, 15] with the efficacy of treatment in ITP patients. However, to our knowledge, no studies have investigated the relationship between these predictors and the therapeutic efficacy of rhTPO in pregnant patients with ITP. Therefore, this study aimed to demonstrate the clinical effectiveness of rhTPO in pregnancy, as well as the haematological predictors of response, among pregnant patients with ITP to provide better treatment strategies for such patients who do not respond to first-line treatment.

## Methods

### Patients

**Fig. 1:**
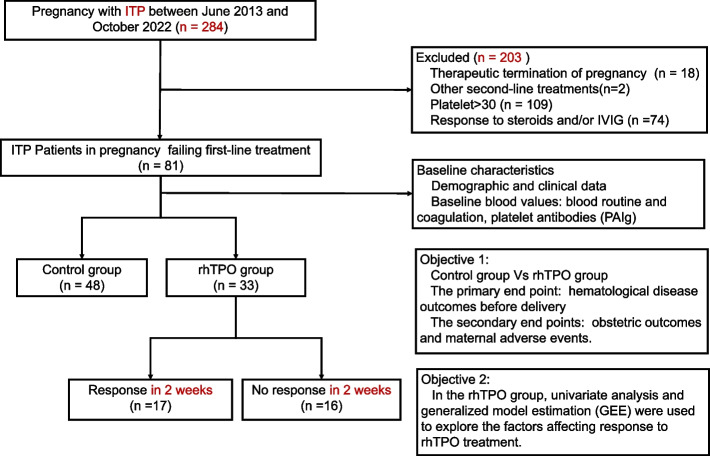
Flow diagram of the study

The clinical data of 284 pregnant women with ITP who were treated and delivered at the Fujian Medical University Union Hospital from June 2013 to October 2022 were collected. ITP was diagnosed based on international consensus [6, 16]. Patients comprised those with ITP diagnosed during and before pregnancy. The inclusion criteria were as follows: (1) Diagnosis of primary immune thrombocytopenia; (2) a platelet count less than 30 × 10^9^/L; and (3) failure to respond to treatment with IVIG and corticosteroids. The exclusion criteria were (1) therapeutic termination of pregnancy (*n* = 18); (2) other second-line ITP-specific treatments (*n* = 2); (3) platelets > 30 × 10^9^/L (*n* = 109); and (4) response to steroids and/or IVIG (*n* = 74). These eligibility criteria identified 81 patients for analysis, 33 of whom received rhTPO treatment (rhTPO group) while 48 did not (control group) (Fig. 1). Informed consents to treatment were obtained by all patients or their guardians. This study was approved by the Ethics Committee of the Fujian Medical University Union Hospital (Approval No: 2023KY026).

### Treatment methods

The patients’ blood was examined every 1–2 weeks, and haemorrhagic manifestations were observed.

rhTPO (TPIAO™, Shenyang Sunshine Pharmaceutical Co., Ltd.) (300 U/kg), was subcutaneously injected once daily for 14 days. When a response was observed, patients thereafter underwent individualised maintenance therapy up to delivery. In the absence of a response after a fortnight, treatment was terminated. Treatment with rhTPO was discontinued when the platelet count increased to 100 × 10^9^/L. Patients received the next cycle of therapy when their platelet count was less than 20–30 × 10^9^/L during pregnancy or less than 50 × 10^9^/L near delivery [4]. All patients were managed by the haematology physician and obstetrician, and an individualized treatment of rhTPO was determined according to the patients’ gestational age, platelet counts, and bleeding tendency.

Platelet transfusions may be used in significant bleeding cases with a platelet count < 10 × 10^9^/L or may be considered if delivery is approaching with platelet counts still below the recommended level after treatment. Caesarean sections and vaginal delivery require a platelet count of ≥ 50 × 10^9^/L. Epidural anaesthesia is generally considered safe at a count of ≥ 70 × 10^9^/L [1, 6]. The survival time of transfused platelets is 1–4 h; thus, platelet transfusions should be performed as close to the time of caesarean section as possible or during the active phase of labour [1, 6, 7].

Delivery was planned after 38–39 weeks of gestation for those who responded to treatment and after 37 weeks for those who did not. Termination of pregnancy can be considered after 34 weeks of gestation when standard treatment failed, and a progressive platelet decline (< 10 × 10^9^/L) and bleeding tendency were observed. The mode of delivery should be considered based on therapeutic effects and obstetrical indications.

The platelet count of the newborns was monitored 3–4 times every other day after birth, and bleeding symptoms were observed.

### Observational indices and definitions

Using electronic medical records and institutional databases, we obtained the baseline demographic, clinical, and blood parameters of all participants. The primary endpoint of this study was haematological disease outcomes before delivery. The secondary endpoints were obstetric outcomes and maternal adverse events. In the rhTPO group, univariate analysis and generalised estimating equation (GEE) were used to explore factors affecting the response to rhTPO treatment (Fig. 1).

Haematological disease outcomes included maternal platelet count, bleeding symptoms, and platelet transfusion rate before delivery.

Obstetric outcomes included gestational weeks of delivery, mode of delivery, postpartum haemorrhage (PPH), and foetal or neonatal complications, including premature birth, low birth weight, neonatal platelets < 100 × 10^9^/L, Apgar score ≤ 7 at 1 min, malformation, stillbirth, and neonatal death.

Maternal adverse events included maternal liver or renal function impairment and thrombosis.

Platelet counts in the first and second weeks after rhTPO treatment were collected in the rhTPO group. The definitions of maternal treatment responses in terms of platelet count were as follows: (1) no response, less than double the baseline count or lower than 30 × 10^9^/L; and (2) response, at least a platelet count of 30 × 10^9^/L or double the baseline count [17]. Response rate was defined as the number of responders within 2 weeks divided by the number of treated patients.

The bleeding score was determined based on Chinese guidelines for treating and diagnosing ITP [17, 18].

### Statistical analyses

Continuous variables corresponding to a normal distribution were expressed as means ± standard deviations and compared using the independent sample t-test; otherwise, medians with interquartile range (IQR) were reported and compared using the Mann–Whitney U test. Categorical variables were compared with χ^2^ or Fisher’s exact test. The treatment response of rhTPO in pregnant women was tracked continuously for 2 weeks, and the data were longitudinal; therefore, univariate logistic regression and GEE analysis were used to identify the factors influencing the response to rhTPO treatment. A robust standard error was used to improve the robustness of the estimation. Parameters with *P*-values < 0.2 in univariate analyses were included in multivariable GEE analyses. Statistical analyses were conducted using SPSS version 24.0 and Stata version 17.0. The *P*-value of all hypothesis tests was 0.05, with a two-sided test.

## Results

### Baseline characteristics

The median (IQR) maternal age was 30 (25–33) years, and the mean body mass index (BMI) was 24.5 ± 3.1 kg/m^2^. Among the patients, forty-seven (58%) were nulliparous and 34 (42%) multiparous. Sixty-five (80.2%) women had a history of ITP before pregnancy, and 16 (19.8%) had no history of ITP before pregnancy. Among them, 48 patients in the control group did not receive other treatments, and 33 patients in the rhTPO group received rhTPO treatment. The baseline characteristics between the aforementioned groups were comparable (all *P* > 0.05) (Table 1). rhTPO treatment was started at 24–35 weeks of gestation. Four mothers received rhTPO treatment from the second trimester, with a platelet count of 1–8 × 10^9^/L and bleeding tendencies, and 29 received rhTPO treatment from the third trimester. All 17 responders maintained their response until delivery. The median time of rhTPO exposure during pregnancy was 3.6 (2–4) weeks for responders.

**Table 1:** Maternal baseline characteristics

Variables	Control group (*n* = 48)	rhTPO group (*n* = 33)	Statistical value	*P*
Age	30 (27.3, 32)	29 (26, 30)	-1.352^a^	0.176
BMI (kg/m^2^)	24.4 ± 2.3	24.6 ± 4.0	-0.407^b^	0.685
Monthly income (CNY)			1.077^c^	0.584
< 5000	13 (27.1)	9 (27.3)		
5000–10000	30 (62.5)	18 (54.5)		
> 10000	5 (10.4)	6 (18.2)		
Number of births, n (%)			3.057^c^	0.217
0	25 (52)	22 (67)		
1	19 (40)	7 (21)		
2	4 (8)	4 (12)		
Diagnosis of ITP, n (%)			0.087^c^	0.768
Before pregnancy	38 (79)	27 (82)		
In pregnancy	10 (21)	6 (18)		
pre-pregnancy platelet(× 10^9^/L)	38 (29.3, 73.8)	45 (37, 64)	-1.491^a^	0.136
Platelet antibodies, n (%)			0.320^c^	0.572
No	8 (17)	4 (12)		
Yes	40 (83)	29 (88)		
Obesity (BMI≧30)			NA	0.393^d^
No	46 (95.8)	30 (90.9)		
Yes	2 (4.2)	3 (9.1)		
Diabetes			NA	0.393^d^
No	46 (95.8)	30 (90.9)		
Yes	2 (4.2)	3 (9.1)		
Hypertension			NA	0.393^d^
No	46 (95.8)	30 (90.9)		
Yes	2 (4.2)	3 (9.1)		
Medications, n (%)			NA	0.683^d^
No	45 (89.6)	30 (90.9)		
Yes	3 (10.4)	3 (9.1)		
Baseline bleeding score, n (%)			0.526^c^	0.468
0	36 (75)	27 (81.8)		
1	9 (18.8)	1 (3)		
2	3 (6.3)	5 (15.2)		
Baseline blood values				
Platelets(× 10^9^/L)	18.6 ± 7.6	15 ± 8.9	1.970^b^	0.052
White cell count (× 10^9^/L)	6.6 (4.7, 10.8)	7.3 (5.4, 11.5)	-0.745^a^	0.456
Neutrophil (%)	65.9 (52.1, 77.9)	71.7 (55.5, 78.5)	-0.971^a^	0.332
Lymphocyte (%)	26.8 (15.9, 38.7)	19.6 (14.6, 29.2)	-1.216^a^	0.224
Hemoglobin (g/L)	105.5 (90, 116.8)	107 (90.5, 115)	-0.125^a^	0.901
Hematocrit (%)	31 (25.9, 33)	31 (24, 35)	-0.644^a^	0.519
MPV (fL)	12.5 ± 2.3	12 ± 2.1	0.963^a^	0.338
PDW (%)	15 (13, 18.9)	15.3 (14.6, 20.8)	-0.870^a^	0.384
P-LCR (%)	38.9 ± 11.7	37.7 ± 9.2	0.478^b^	0.634
NLR	0.7 (0.4, 1.4)	0.6 (0.3, 1.2)	-1.447^a^	0.148
PLR	11.4 (7, 15.5)	9.2 (4.4, 17.6)	-1.043^a^	0.297
Prothrombin time (s)	12.3 ± 0.8	12.2 ± 0.6	0.919^b^	0.361
Activated partial thromboplastin time(s)	29.8 ± 3.6	31.3 ± 4.2	-1.683^b^	0.096
Fibrinogen (g/L)	4.2 ± 1.1	4.2 ± 1.1	0.076^b^	0.940
Dimer, μg/ml	2.5 (1.4, 5.5)	2.2 (1.4, 5.4)	-0.005^a^	0.996

*Abbreviations*: *BMI* body mass index, *NA* not applicable, *MPV* mean platelet volume, *PDW* platelet distribution width, *P-LCR* platelet-large cell ratio, *NLR* neutrophil–lymphocyte ratio, *PLR* platelet-lymphocyte ratio

^a^Mann-Whitney U Test

^b^Independent-Samples Test

^c^Chi-square test

^d^Fisher exact probability test

### Haematological disease outcomes before delivery

The platelet counts before delivery in the rhTPO and control groups were 42 (21.5–67.5) × 10^9^/L and 25 (19–29) × 10^9^/L, respectively, with a significant difference (*P* = 0.002). The rate of bleeding (bleeding score ≥ 1) (6.1% vs. 25%, *P* = 0.027) and platelet transfusion (57.6% vs. 97.9%, *P* < 0.001) before delivery were significantly lower in the rhTPO group than in the control group (Table 2). In whole cohort, 68 patients had platelet count less than 50 × 10^9^/L before delivery, of whom 66 received platelet transfusions before either vaginal delivery or caesarean section. Twenty patients in the rhTPO group exhibited platelet counts below 50 × 10^9^/L before delivery, of whom 4 displayed a response to treatment. In the rhTPO group, a total of 19 patients received platelet transfusions before delivery, comprising 16 non-responders (100%, 16/16) and 3 (17.6%, 3/17) responders (Table 4). Among the 17 responders, 13 had a platelet count above 50 × 10^9^/L before delivery, and 7 had a platelet count above 70 × 10^9^/L before delivery.

**Table 2:** Hematological disease outcomes before delivery between two group

Variables	Control group (*n* = 48)	rhTPO group (*n* = 33)	Statistical value	*P*
Before delivery				
Platelets (× 10^9^/L)	25 (19, 29)	42 (21.5, 67.5)	-3.049^a^	0.002
Bleeding score, n (%)			4.906^b^	0.027
0	36 (75)	31 (93.9)		
1	9 (18.8)	2 (6.1)		
2	3 (6.3)	0 (0)		
Platelet transfusion, n (%)			21.091^b^	< 0.001
No	1 (2.1)	14 (42.4)		
Yes	47 (97.9)	19 (57.6)		

Platelets before delivery: platelet counts before platelet transfusion before delivery

^a^Mann-Whitney U Test

^b^Chi-square test

### Obstetric outcomes

Gestational weeks of delivery were significantly longer in the rhTPO group than in the control group (37.6 [37–38.4] vs. 37.1 [37–37.2], *P* = 0.001). No significant differences were observed in delivery mode, PPH rate, rate of foetal or neonatal complications, or complication type (*P* > 0.05, both; Table 3). No foetal malformations, stillbirths, or neonatal deaths were observed in both groups. Twenty-three (28.3%) cases of neonatal thrombocytopenia were identified, four of which revealed skin petechiae; no case of intracranial haemorrhage (ICH) was observed. There were no statistically significant differences observed in neonatal platelet counts on days 1, 3, and 5 between the two groups (all *P* > 0.05; Table 3). In whole cohort, of 14 preterm deliveries, 2 were found to be associated with ITP. Deliveries in these cases took place at 35–36 weeks due to ineffective treatment and platelet counts below 10 × 10^9^/L, accompanied with bleeding tendency. Three of the 60 patients who underwent caesarean section were under epidural anaesthesia, while the remaining 57 patients were operated under general anaesthesia.

**Table 3:** Obstetric outcomes between two group

Variables	Control group (*n* = 48)	rhTPO group (*n* = 33)	Statistical value	*P*
Gestational weeks of delivery (weeks)	37.1 (37, 37.2)	37.6 (37, 38.4)	-3.356^a^	0.001
Mode of delivery			1.591^b^	0.207
Vaginal delivery, n (%)	10 (20.8)	11 (33.3)		
Cesarean section, n (%)	38 (79.2)	22 (66.7)		
Postpartum hemorrhage, n (%)			0.188^b^	0.665
No	41 (85.4)	27 (81.8)		
Yes	7 (14.6)	6 (18.2)		
Postpartum hemorrhage (ml)	500 (400, 800)	500 (234, 680)	-0.697^a^	0.486
Fetal or neonatal complications, n (%)			0.313^b^	0.576
No	32 (66.7)	20 (60.6)		
Yes	16 (33.3)	13 (39.4)		
Premature, n (%)			0.031^b^	0.859
No	40 (83.3)	27 (81.8)		
Yes	8 (16.7)	6 (18.2)		
Low birth weight (< 2.5 kg), n (%)			0.405^b^	0.524
No	43 (89.6)	28 (84.8)		
Yes	5 (10.4)	5 (15.2)		
Neonatal platelets < 100 × 10^9^/L, n (%)			0.100^b^	0.752
No	35 (72.9)	23 (69.7)		
Yes	13 (27.1)	10 (30.3)		
Apgar score≦7 at 1 min, n (%)			NA	0.511^c^
No	46 (95.8)	33 (100)		
Yes	2 (4.2)	0 (0)		
Neonatal platelets on day 1(× 10^9^/L)	150 (106.8, 181)	123 (90.5, 156)	-1.558^a^	0.119
Neonatal platelets on day 3(× 10^9^/L)	151 (87.8, 188)	121 (72, 162)	-1.761^a^	0.078
Neonatal platelets on day 5(× 10^9^/L)	170 (107.5, 204)	165 (113, 183)	-1.077^a^	0.282

Postpartum hemorrhage was defined as a blood loss of ≥ 500 ml from the genital tract for vaginal deliveries and ≥ 1000 ml for caesarean sections, within 24 h of delivery

Fetal or neonatal complications: including premature, low birth weight, neonatal platelets < 100 × 10^9^/L, Apgar score≦7 at 1 min, fetal malformations, stillbirth and neonatal death

*Abbreviations*: *NA* not applicable

^a^Mann-Whitney U Test

^b^Chi-square test

^c^Fisher exact probability test

In the rhTPO group, the median gestational weeks of delivery for responders was 38.3 (38–39.3), while the median gestational weeks of delivery for non-responders was 37 (36–37.4) (*P* < 0.001). Caesarean delivery rates were 35.3% (6/17) and 100% (16/16) among responders and non-responders, respectively (*P* < 0.001). There was no significant difference in PPH, PPH rates and premature delivery rates between responders and non-responders (all *P* > 0.05; Table 4).

**Table 4:** Outcomes in rhTPO group

Variables	No response (*n* = 16)	Response (*n* = 17)	Statistical value	*P*
Platelets before delivery (× 10^9^/L)	19 (17.3, 25)	68 (48.5, 112)	-4.909^a^	< 0.001
Platelet transfusion, n (%)			NA	< 0.001^b^
No	0 (0)	14 (82.4)		
Yes	16 (100)	3 (17.6)		
Gestational weeks of delivery (weeks)	37 (36, 37.4)	38.3 (38, 39.3)	-4.151^a^	< 0.001
Mode of delivery			NA	< 0.001^b^
Vaginal delivery, n (%)	0 (0)	11 (64.7)		
Cesarean section, n (%)	16 (100)	6 (35.3)		
Postpartum hemorrhage, n (%)			NA	0.085^b^
No	11 (68.8)	16 (94.1)		
Yes	5 (31.30)	1 (5.9)		
Postpartum hemorrhage (ml)	600 (350, 1000)	400 (159, 550)	-1.835^a^	0.066
Premature, n (%)			NA	0.085^b^
No	11 (68.8)	16 (94.1)		
Yes	5 (31.3)	1 (5.9)		

Platelets before delivery: platelet counts before platelet transfusion before delivery

Postpartum hemorrhage was defined as a blood loss of ≥ 500 ml from the genital tract for vaginal deliveries and ≥ 1000 ml for caesarean sections, within 24 h of delivery

*Abbreviations*: *NA* not applicable

^a^Mann-Whitney U Test

^b^Fisher exact probability test

### Maternal adverse events

No liver or renal function impairment or thrombosis cases were observed in the rhTPO group. One pregnant woman in the control group developed intracranial venous sinus thrombosis with multiple ICH after a caesarean section and was cured with no sequelae.

### Factors predicting response to rhTPO treatment

**Fig. 2:**
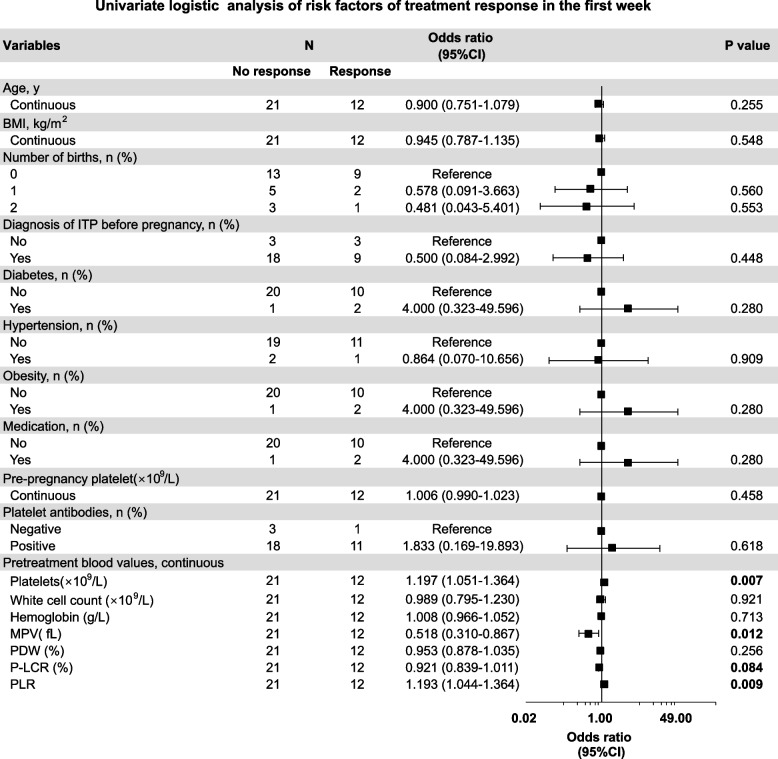
Screening for the factors associated with treatment response (in the first week). Forest plot based on univariate analysis for maternal baseline characteristics and baseline blood values as potential risk factors. BMI: body mass index; MPV: mean platelet volume; PDW: platelet distribution width; P-LCR: platelet-large cell ratio; PLR: platelet-lymphocyte ratio

**Fig. 3:**
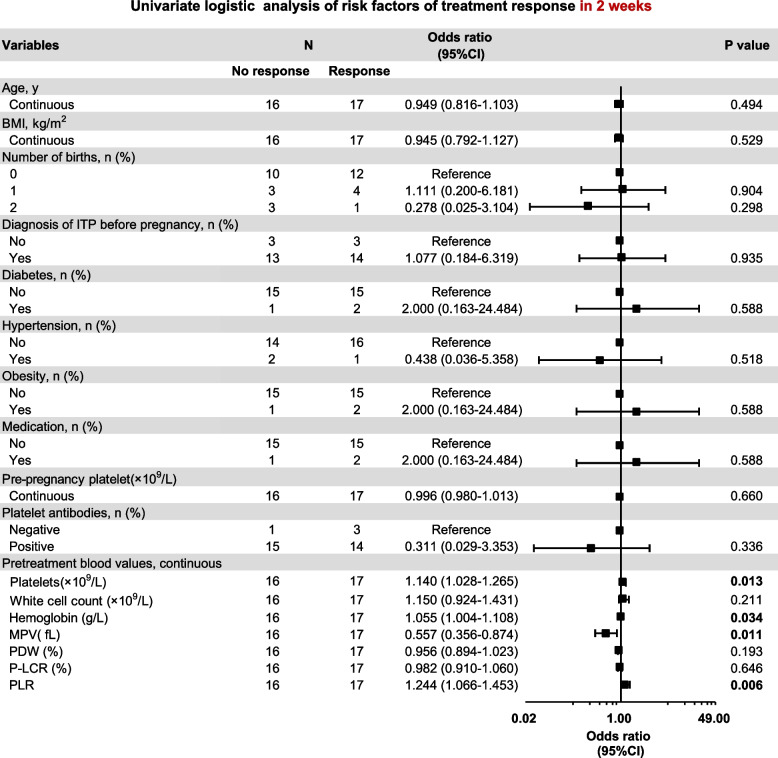
Screening for the factors associated with treatment response (in 2 weeks). Forest plot based on univariate analysis for maternal baseline characteristics and baseline blood values as potential risk factors. BMI: body mass index; MPV: mean platelet volume; PDW: platelet distribution width; P-LCR: platelet-large cell ratio; PLR: platelet-lymphocyte ratio

In the rhTPO group, 12 patients responded in the first week, and a further 5 patients responded in the second week, with a response rate of 51.5% (17/33) in 2 weeks. The response to rhTPO in the first week was significantly correlated with baseline platelet count, mean platelet volume (MPV), platelet-large cell ratio (P-LCR), and platelet-to-lymphocyte ratio (PLR) (Fig. 2). The response to rhTPO in the 2 weeks was significantly related to the baseline platelet count, haemoglobin level, MPV, platelet distribution width (PDW), and PLR (Fig. 3). Finally, GEE analysis revealed that a higher baseline MPV was associated with a lower response to rhTPO treatment (odds ratio [OR]: 0.522, *P* = 0.002), and a higher baseline PLR was associated with a higher response to rhTPO treatment (OR: 1.214; *P* = 0.025) (Table 5).

**Table 5:** GEE result with robust standard error

Variables	Odds ratio	Robust std.err	z	*P* >|z|	OR 95% conf.interval	
Baseline blood values						
Platelets	1.039	0.084	0.480	0.631	0.888	1.217
Hemoglobin	1.033	0.030	1.130	0.259	0.976	1.093
MPV	0.522	0.111	-3.050	0.002	0.344	0.793
PDW	0.964	0.029	-1.220	0.222	0.908	1.023
P-LCR	0.981	0.047	-0.390	0.695	0.893	1.078
PLR	1.214	0.105	2.240	0.025	1.024	1.438

*MPV* mean platelet volume, *PDW* platelet distribution width, *P-LCR* platelet-large cell ratio, *PLR* platelet-lymphocyte ratio

## Discussion

Our data demonstrated that, compared with the control group, the rhTPO group had significantly increased platelet levels, ameliorated bleeding, decreased platelet transfusions before delivery, and extended gestational weeks of delivery. No significant differences were observed in the rates of caesarean section, PPH, foetal or neonatal complications, or their types between both groups. No cases of liver or renal function impairment or thrombosis were observed in the rhTPO group. Patients with a lower baseline MPV and higher baseline PLR were more responsive to rhTPO treatment. Thus, rhTPO is a therapeutic option for pregnant patients with ITP who do not respond to steroids and/or IVIG.

We investigated how pregnant patients with ITP for whom first-line treatment has failed could be managed. In our study, the rhTPO group had significantly increased platelet counts, alleviated bleeding, and decreased platelet transfusions rate before delivery compared with the control group. These results suggest that rhTPO is effective for ITP treatment during pregnancy when first-line therapy fails. However, the rate of platelet transfusion before delivery in the rhTPO group remained high at 57.6% (19/33). This can be attributed to two possible causes. First, the study included patients with platelet counts below 30 × 10^9^/L who had failed to respond to first-line treatment. Secondly, in the rhTPO group, the response rate to rhTPO treatment was 51.5% (17/33). Consequently, all 16 non-responders (100%, 16/16) and 3 responders (17.6%, 3/17) underwent platelet transfusion before delivery in accordance with the international consensus as their platelet counts remained below the recommended safe delivery threshold (50 × 10^9^/L). Eighty-four point two percent (16/19) of platelet transfusions were given to those non-responders. In contrast, responders exhibited a notable decrease in transfusion rates compared with non-responders (*P* < 0.001). Accordingly, it is imperative to conduct further analysis on clinical predictors linked to the response of rhTPO treatment, to improve platelet counts before delivery and decrease the platelet transfusion rate before delivery. In this study, 87.9% (29/33) of women started received rhTPO therapy in the third trimester, reducing drug exposure and allowing enough time to improve platelet counts reduces the risk of bleeding during planned or unplanned caesarean section or vagina delivery. In the case of shortage of blood products, treatment with rhTPO can alleviate low platelet count, improving the safety of pregnancy and delivery.

In addition to effectiveness, obstetric outcomes were also considered. Compared with the control group, the rhTPO group had significantly extended gestational weeks of delivery. We observed a decrease in the caesarean section rate in the rhTPO group (79.2% in the control group vs. 66.7% in the rhTPO group). However, the difference between both groups was insignificant, possibly due to the limited sample size. In this study, both groups of patients had a high rate of caesarean sections. In addition to obstetric indications, 48.3% of caesarean sections were performed owing to low platelet counts (48.3%, 29/60). The caesarean section rates among pregnant patients with ITP are associated with platelet count, response to drug treatment, and cervical maturity. In our study, 64 patients (64/81,79%) exhibited a platelet count before delivery below 30 × 10^9^/L, including 48 patients in the control group and 16 non-responders in the rhTPO group. Of 64 patients, 29 underwent caesarean section due to thrombocytopenia, characterized by platelet counts before delivery below 20 × 10^9^/L and cervical immaturity. When platelet count was below 20 × 10^9^/L before delivery, especially with a bleeding tendency, as well as cervical immaturity, caesarean section can be considered because of the short survival time of the transfused platelets. Caesarean section has a short delivery time, the safe threshold for delivery (50 × 10^9^/L) can be maintained by platelet transfusion. Responders had a lower rate of caesarean section than had non-responders (35.3% vs. 100%, *P* < 0.001). Hence, it is important to explore potential indicators that could forecast the response to rhTPO treatment, to increase and maintenance platelet counts before delivery and decrease the rate of caesarean section. According to the National Maternal and Child Health Statistics, the caesarean section rate in China exhibited a rate of 28.8%–36.7% between 2008 and 2018 [19]. The caesarean section rate observed in our institution between 2018 and 2022 ranged from 41.2% to 45.6%, with primary reasons comprising obstetric indications and presence of severe comorbidities and complications. Relevant literatures at home and abroad also showed that [2, 20] patients with thrombocytopenia during pregnancy exhibit a comparatively elevated incidence of caesarean section. The lower the platelets, the higher the rate of caesarean section [20].

ITP during pregnancy is a high-risk factor for PPH [2]; however, no differences were observed in PPH incidence between both groups. This result may be explained by the effect of platelet transfusion before delivery. Huang et al. [5] revealed that PPH in patients with ITP is related to antepartum platelet transfusion and platelet count. The causes of the 13 cases (16%, 13/81) of PPH in our study were as follows: acute perineal laceration (*n* = 1), uterine incision laceration (*n* = 1), uterine fatigue (*n* = 10), and placental factors (*n* = 1) possibly related to obstetric factors such as uterine atony and abnormal placentation. Thus, to prevent PPH, platelet transfusion to elevate platelets at delivery to the “safe” level and good contractions are key while avoiding injury and adequate haemostasis. Overall, foetal and neonatal outcomes were favourable, and no significant differences were observed in foetal or neonatal complications or type between both groups. Patients in the rhTPO group commencing rhTPO administration from 24 to 35 weeks of gestation exhibited no additional adverse effects on the foetuses or neonates compared with those in the control group. The incidence of a neonatal platelet count < 100 × 10^9^/L was 28.3% (23/81). These results are similar to those of previous studies [4, 8, 9]. There was no difference in neonatal platelets between both groups, and no cases of neonatal thrombocytosis were observed. This finding provides additional evidence supporting that rhTPO does not cross the placenta. In the present study, only neonatal outcomes at delivery were recorded, and there were no long-term follow-up of neonatal growth and development. In Kong's study [4], a median follow-up of 53 (range, 39–68) weeks revealed no instances of congenital diseases or developmental delays among the newborns.

As rhTPO is a highly specific platelet-stimulating factor, attention should be paid to thrombosis prevention during its use. No thromboembolic events were observed in the rhTPO group; however, one pregnant woman in the control group developed ‘intracranial venous sinus thrombosis with ICH’ 20 days after caesarean section. Patients with ITP are at risk of bleeding and thromboembolism [7, 21, 22]. Therefore, all pregnant women with ITP should receive appropriate thromboembolic prophylaxis [6, 7].

Factors affecting the treatment response to rhTPO were further analysed. GEE analysis revealed that baseline MPV and PLR were the two factors that could affect response to rhTPO treatment. MPV reflects platelet production in the bone marrow. A high MPV indicates high platelet production, and a low MPV indicates low platelet production [23, 24]. A previous study revealed that newly diagnosed adult patients with primary ITP with high baseline MPV were more likely to respond to first-line treatment [12]. However, our results revealed that patients with a lower baseline MPV were more responsive to rhTPO treatment, inconsistent with the results of a previous study. Two possible reasons may explain this finding. First, the population included in this study were pregnant women with ITP who did not respond to first-line treatment, and the population included in the previous study were newly diagnosed adults with ITP. Second, the therapeutic drugs used have different mechanisms of action. Steroid and/or IVIG treatments inhibit platelet destruction, whereas rhTPO promotes platelet production. A low MPV indicates insufficient platelet generation [25–27]. rhTPO stimulates the formation and differentiation of bone marrow megakaryocytes, which subsequently produce platelets [28, 29]. Thus, patients with lower baseline MPV were more responsive to rhTPO treatment. PLR is an inflammatory biomarker derived from platelet and lymphocyte indices. In our study, pregnant patients with ITP who failed first-line therapy with higher baseline PLR levels were more responsive to rhTPO. This result is consistent with that of a previous study [14]. In addition to its direct role in stimulating platelet production by megakaryocytes, rhTPO has demonstrated additional effects on immune regulation [10, 11]. These findings suggest that pregnant patients with ITP with low baseline MPV and high baseline PLR respond better to rhTPO treatment when first-line treatment fails. To our knowledge, our study is the first to analyse the predictors of response to rhTPO treatment for ITP during pregnancy.

There is no consensus on second-line treatment options. TPO-RAs are a small molecule that can cross the placenta and may cause neonatal thrombocytosis [30]. International [6] and Chinese [31] consensuses state that these TPO-RA drugs should be used only in extremely severe cases, when other treatments fail or are not available, and only in the third trimester, as a means to increase platelet counts before delivery. Rituximab can cross the placenta [32], and the response may be slow (up to 8 weeks). If administered in the third trimester, rituximab may increase foetal hypogammaglobulinaemia and affect neonatal vaccine use [33, 34]. Azathioprine and cyclosporine also have a slow response, taking weeks to increase platelets, and their safety data are almost entirely based on the treatment of other diseases [35, 36]. According to the current study, rhTPO may be a viable alternative for pregnant women with ITP who failed first-line treatment.

Our study had some limitations. First, this was a retrospective cohort study, and selection bias could not be avoided. Second, long-term follow-up data on neonatal outcomes are not yet available. Furthermore, owing to the relatively low ITP incidence during pregnancy, the sample size was small despite this study including 10-year cases in a tertiary hospital with a blood disease referral centre. However, to our knowledge, this is the first cohort study that compared rhTPO treatment with no treatment in pregnant women with ITP in whom first-line treatment failed, serving as a basis for prospective clinical trials.

### Conclusions

In conclusion, our limited data suggest that rhTPO may be an effective and safe treatment option for pregnant patients with ITP in whom first-line treatment fails and may slightly prolong the gestational age of delivery. Patients with a low baseline MPV and high baseline PLR may be more responsive to rhTPO treatment. Our study provides clinical evidence that supports the use of rhTPO in pregnant patients with ITP. However, the optimal dosing regimen needs to be explored to optimise treatment. Further multi-centre prospective clinical trials are warranted.

## Data Availability

The data is available from the corresponding author upon reasonable request.

## Abbreviations

rhTPO: Recombinant human thrombopoietin
ITP: Immune thrombocytopenia
GEE: Generalised estimating equation
MPV: Mean platelet volume
OR: Odds ratio
PLR: Platelet-to-lymphocyte ratio
IVIG: Intravenous immunoglobulins
TPO: Thrombopoietin
TpoR: Thrombopoietin receptor
PPH: Postpartum haemorrhage
BMI: Body mass index
ICH: Intracranial haemorrhage
